# Supplementary material for: Sex-specific role for the long noncoding RNA Pnky in mouse behavior
Source: Nat Commun. 2024 Aug 12;15:6901. doi: 10.1038/s41467-024-50851-7 (PMC11319455; doi:10.1038/s41467-024-50851-7)
Supplement: Supplementary file 1 — Supplementary Information [file 41467_2024_50851_MOESM1_ESM.pdf]

Supplementary Information

Sex-specific role for the long noncoding RNA *Pnky* in mouse behavior

Parna Saha, Rebecca E. Andersen, Sung Jun Hong, Eugene Gil, Jeffrey Simms, Hyeonseok Choi, Daniel A. Lim*.

This PDF file includes:

Supplementary Figures 1-12

* To whom correspondence should be addressed. Email: daniel.lim@ucsf.edu

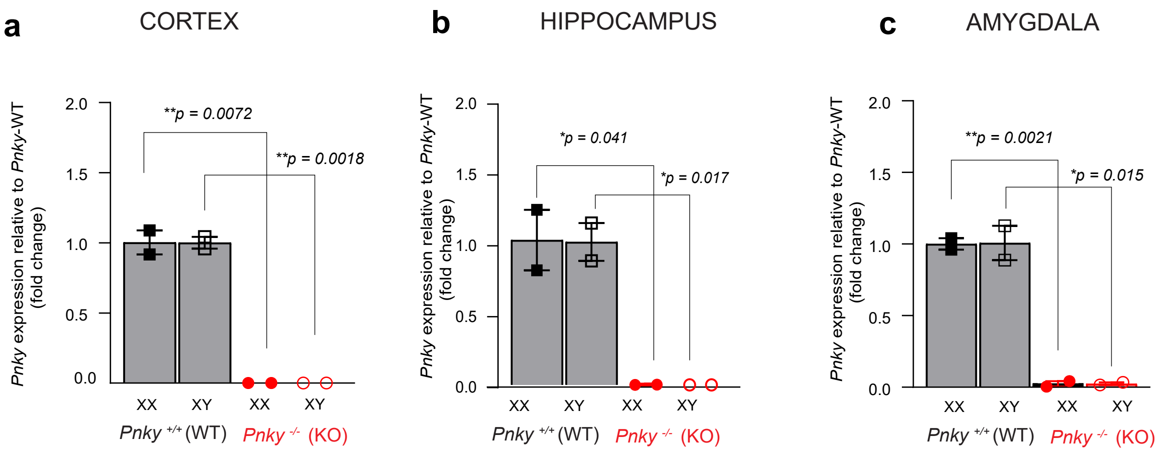

Supplementary Fig. 1: RT-qPCR analyses confirm the absence of *Pnky* RNA in *Pnky*-KO mice.
Pnky RNA expression relative to *Pnky*+/+ mean for respective sex in **a)** cortex, **b)** hippocampus and **c)** amygdala. Quantification: mean ± SEM of biological replicates (n = 2 XX, 2XY mice of each genotype). Statistical analyses: unpaired *t* test (two-tailed). ns, not significant, *$p<0.05$, **$p<0.001$. Source data are provided as a Source Data file.

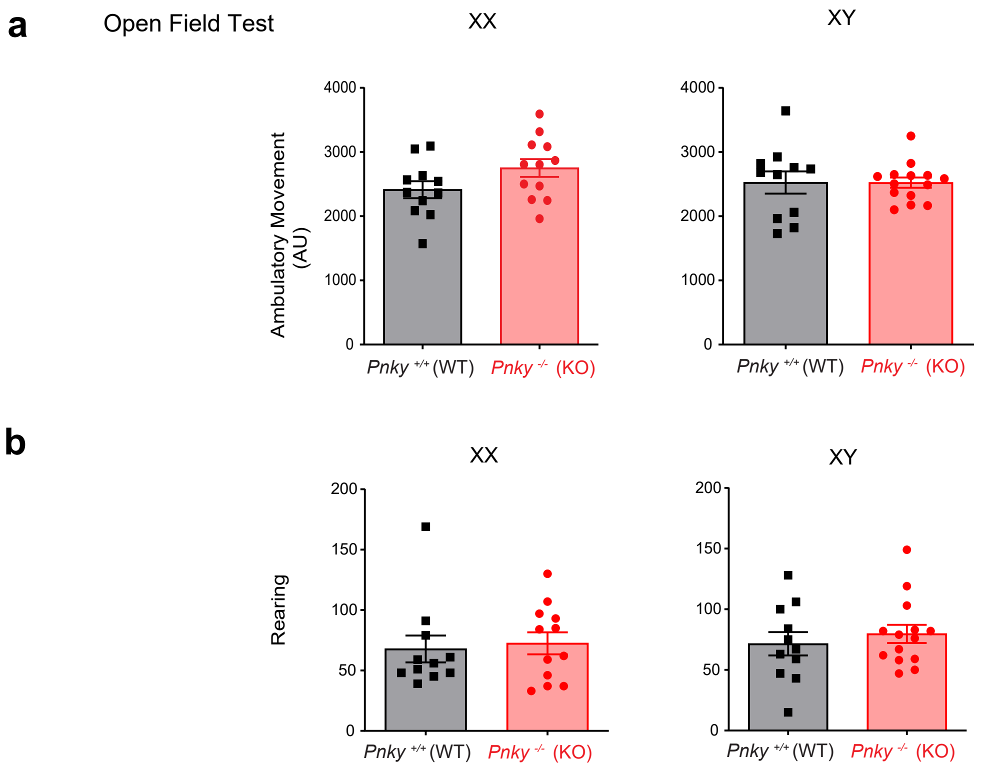

Supplementary Fig. 2: Open field test *Pnky*-WT vs *Pnky*-KO. a) Ambulatory movement in open field test is comparable in both sexes and both genotypes; two-tailed, unpaired Welch's *t* test; $p =$ ns (XX: $p = 0.0933$, XY: $p = 0.9892$). **b)** Rearing behavior shows no significant difference between *Pnky*-WT and *Pnky*-KO animals; two tailed Mann-Whitney test, $p =$ ns (XX: $p = 0.6180$, XY: $p = 0.6185$). n = 11 XX and 11 XY *Pnky*-WT and n = 12 XX and 14 XY *Pnky*-KO mice. Data is represented as mean ± SEM. Source data are provided as a Source Data file.

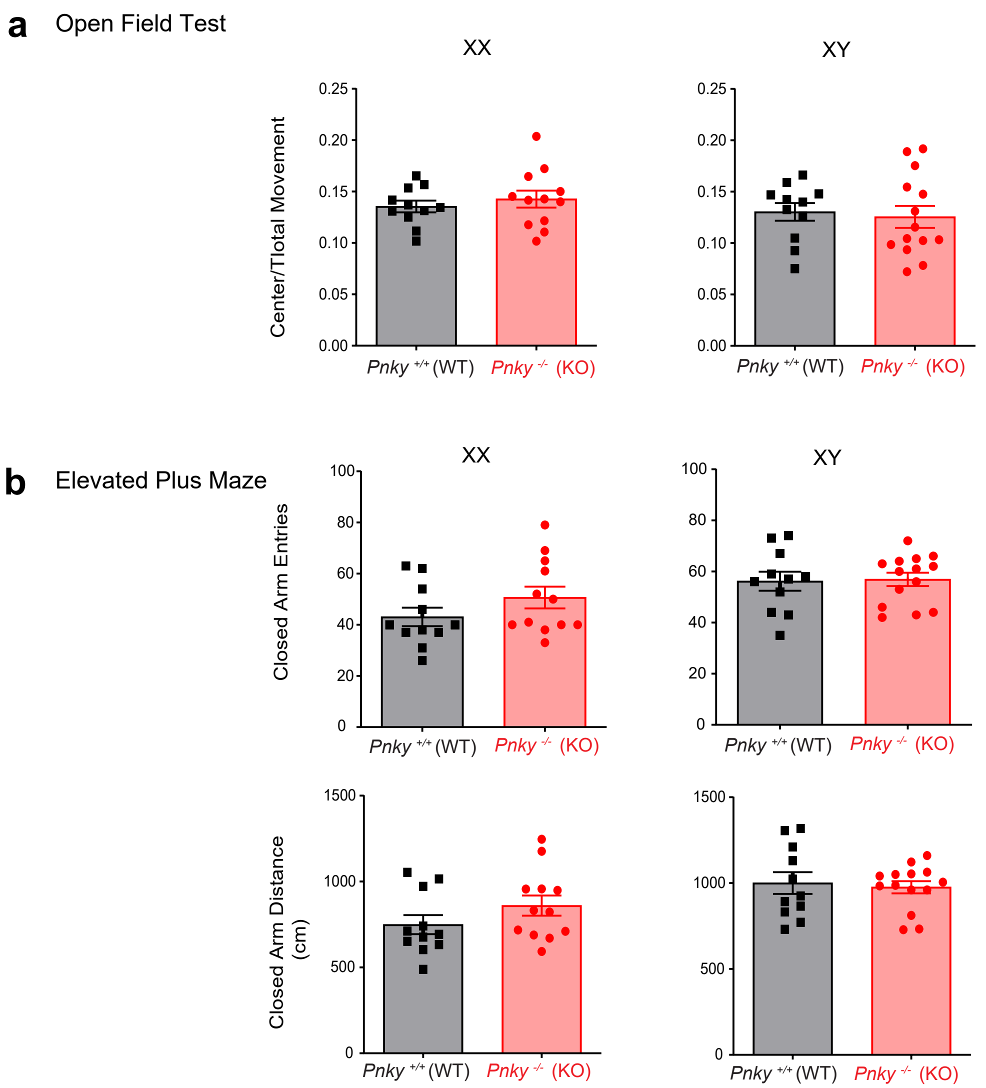

Supplementary Fig. 3: Open field and Elevated Plus Maze test *Pnky*-WT vs *Pnky*-KO. a) *Pnky*-WT and *Pnky*-KO mice have comparable center to total movement ratio in the open field test; Welch's *t* tests, p = ns (XX: $p = 0.4785$, XY: $p = 0.7282$). **b)** In the elevated plus maze test, XX and XY animals show no significant genotype-specific difference in number of entries in the closed arms , Welch's *t* test; p = ns (XX: $p = 0.1880$, XY: $p = 0.8710$) and distance travelled in the closed arms; Welch's *t* tests, p = ns (XX: p =0.1839 , XY: $p = 0.7413$). Unpaired Welch's *t* tests are two-tailed. n = 11 XX and 11 XY *Pnky*-WT and n = 12 XX and 14 XY *Pnky*-KO mice. Data is represented as mean ± SEM. Source data are provided as a Source Data file.

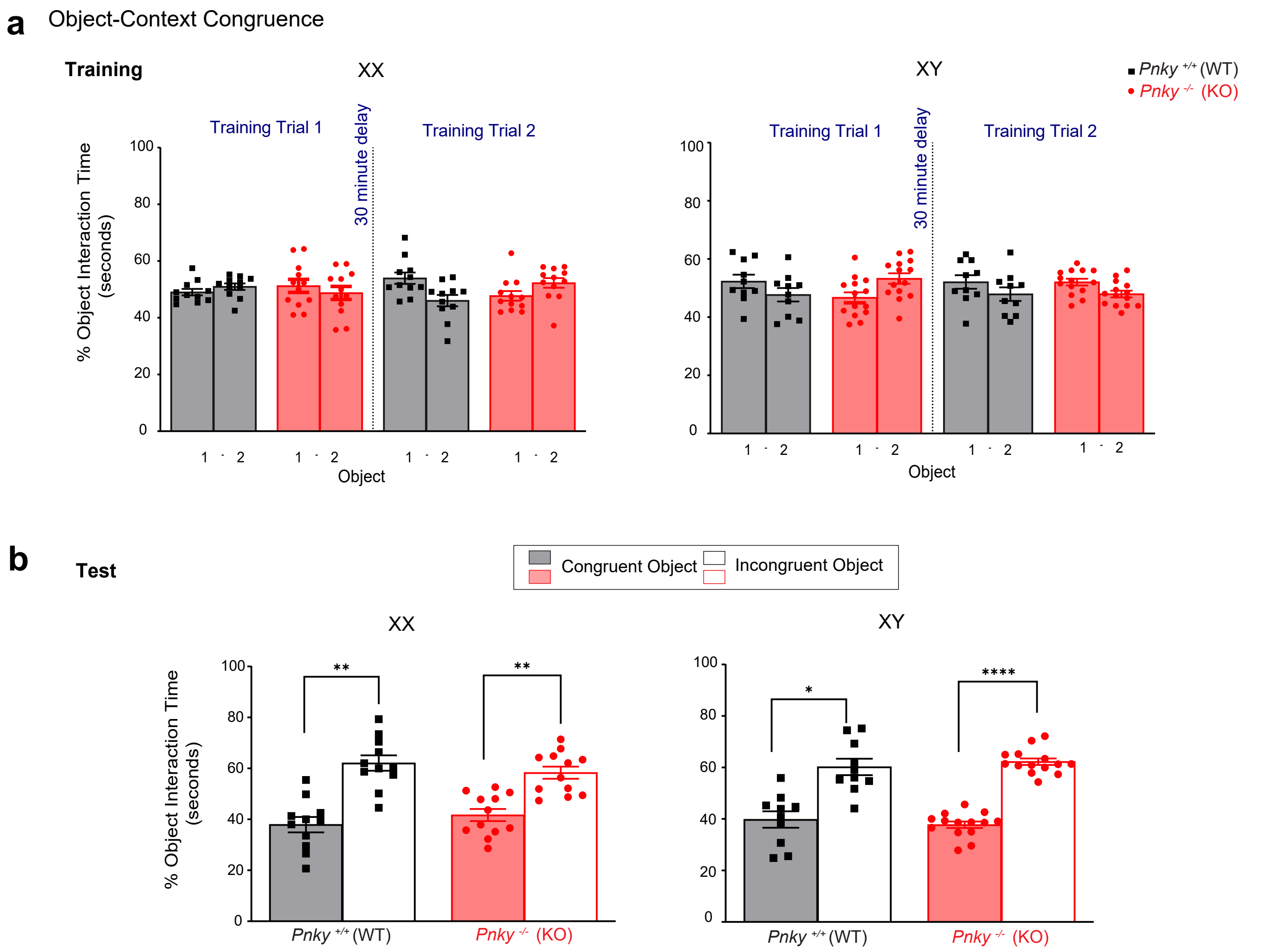

Supplementary Fig. 4: Object congruence context test *Pnky*-WT vs *Pnky*-KO. a) *Pnky*-KO and WT mice did not exhibit significant differences in the two training trials of the object context congruence task, two-tailed, paired *t* tests, p = ns (XX: *Pnky*-WT, Trial 1 $p = 0.1747$, Trial 2 $p = 0.0989$ and *Pnky*- KO, Trial 1 $p = 0.8025$, Trial 2 $p = 0.1683$; XY: *Pnky*-WT, Trial 1 $p = 0.5566$, Trial 2 $p = 0.2797$ and *Pnky*- KO, Trial 1 $p = 0.1564$, Trial 2 $p = 0.2151$. **b)** XX and XY mice of both genotypes show increased preference (% interaction time in seconds) for the incongruent object whereas the difference in interaction time across genotypes is non-significant. Paired *t* test $^{**}p = 0.0025$ for *Pnky*-WT XX mice congruent vs incongruent. Paired *t* test $^{**}p = 0.0051$ for *Pnky*- KO XX mice congruent vs incongruent. Paired *t* test $^{*}p= 0.0109$ for *Pnky*-WT XY mice and $^{****}p <0.0001$ for *Pnky*-KO XY mice congruent vs incongruent. Percent incongruent interaction time was not different between the *Pnky*-WT and *Pnky*-KO of either sex (unpaired Welch's *t*-tests, p = ns (XX: $p= 0.3402$, XY: $p = 0.5665$), $^{*}p < 0.05$, $^{**}p < 0.01$, $^{****}p < 0.0001$, ns = non-significant. Paired *t* tests and Welch's *t* tests are two-tailed. n = 11 XX and 11 XY *Pnky*-WT and n = 12 XX and 14 XY *Pnky*-KO mice. Data is represented as mean ± SEM. Source data are provided as a Source Data file.

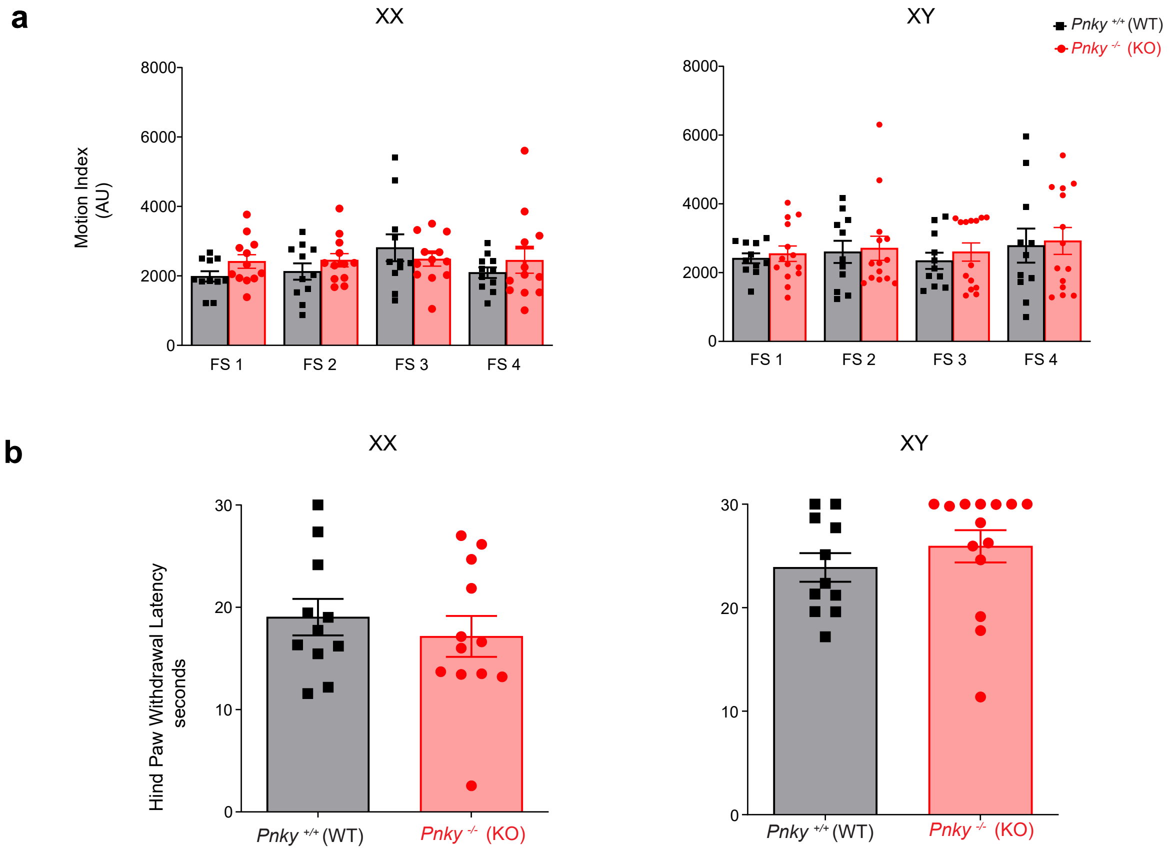

Supplementary Fig. 5: Motion index and Hind paw withdrawal latency test *Pnky*-WT vs *Pnky*-KO. a) In the Motion index test of the Cued Fear conditioning (body movements measured by pixel changes between successive frames in the video recording of freezing behavior to quantify fear strength in response to the stimulus (foot shock (FS)), no significant differences between *Pnky*-WT vs *Pnky*-KO XX and XY animals; multiple unpaired *t* tests, p = ns (FS1- XX: $p = 0.1013$, XY: $p = 0.6437$; FS2- XX: $p = 0.2924$, XY: $p = 0.8330$; FS3- XX: $p = 0.4438$, XY: $p = 0.4893$; FS4- XX: $p = 0.4048$, XY: $p = 0.8291$) **b)** *Pnky*-WT vs *Pnky*-KO XX and XY animals showed no significant difference in hind paw withdrawal latency during the hot-plate test. (For XX genotype Welch's *t* test $p = 0.4880$ and for XY genotype Mann-Whitney test, $p = 0.5658$). n = 11 XX and 11 XY *Pnky*-WT and n = 12 XX and 14 XY *Pnky*-KO mice. Data is represented as mean ± SEM. All Mann-Whitney and Welch's *t* tests are two-tailed. Source data are provided as a Source Data file.

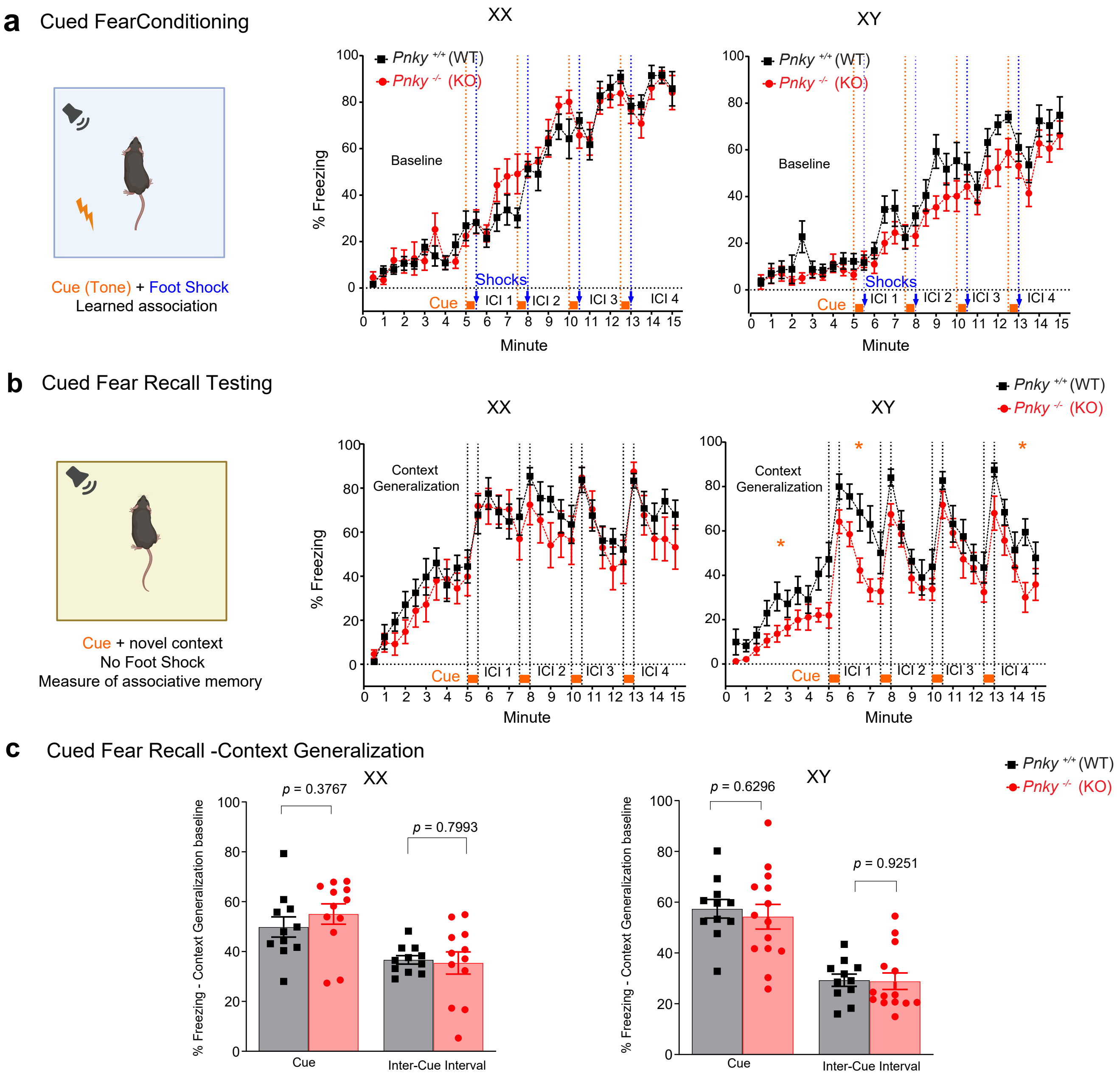

Supplementary Fig. 6: Cued fear conditioning and recall test *Pnky*-WT vs *Pnky*-KO. a) Cued fear conditioning test starts with a 5 min baseline period to measure baseline freezing activity.Then four-30 second 80dB tones that co-terminate with a 2-second, 0.45mA footshock are presented, separated by 120-second inter-cue interval (ICI) during which freezing (% freezing = percentage of time spent (seconds) freezing when conditioned stimulus (CS) is presented during the total duration of the CS) is monitored. A 120 second ICI follows the last footshock. Freezing behavior in *Pnky*-KO mice was comparable to its WT controls (rank summary analysis for XX and XY baseline $p = 0.4001$ and $p = 0.0956$, respectively; multiple t tests during the cue presenta -tions for XX and XY mice, $p = 0.7454$ and $p = 0.2637$, respectively; multiple t tests for XX and XY ICI, $p = 0.5250$ and $p = 0.0762$, respectively) **b)** In the **cued fear recall test**, after a 5 min context generalization period, four tones are delivered as described in conditioning phase. No shock is presented. *Pnky*-WT and *Pnky*-KO XX mice exhibit no significant difference in freezing behavior (linear mixed effects analysis for generalization; multiple t tests during the cue presentations $p = 0.7042$; multiple t tests during the cue presentations p = ns ($p = 0.8970$) linear mixed analysis for ICI 1, 3, and 4, p = ns (ICI 1, $p = 0.6166$; ICI 3, $p = 0.8080$; ICI 4, $p = 0.7950$; rank summary analysis for ICI 2, p = ns (0.6358). *Pnky*-KO XY mice show decreased freezing compared to the WT control in the 5-minute generalization period (rank summary analysis, *$p = 0.0191$), during the cue presentations (multiple t tests, *$p = 0.0301$) and during the ICI (linear mixed effects analysis revealed significant differences for ICI 1 and 4, *$p = 0.0237$ and *$p = 0.0478$,respectively, but not for ICI 2 and 3, $p = 0.4609$ and $p = 0.4674$). **c)** Due to the reduced % freezing in context generalization phase cued fear recall test, when the baseline freezing of context generalization phase is subtracted, the Cued and inter-cue interval phases are non-significant between the *Pnky*-WT vs *Pnky*-KO XY mice (multiple t tests p = ns). n = 11 XX and 11 XY *Pnky*-WT and n = 12 XX and 14 XY *Pnky*-KO mice. Data is represented as mean ± SEM. Source data are provided as a Source Data file. Cartoons in this figure are generated using Biorender.com.

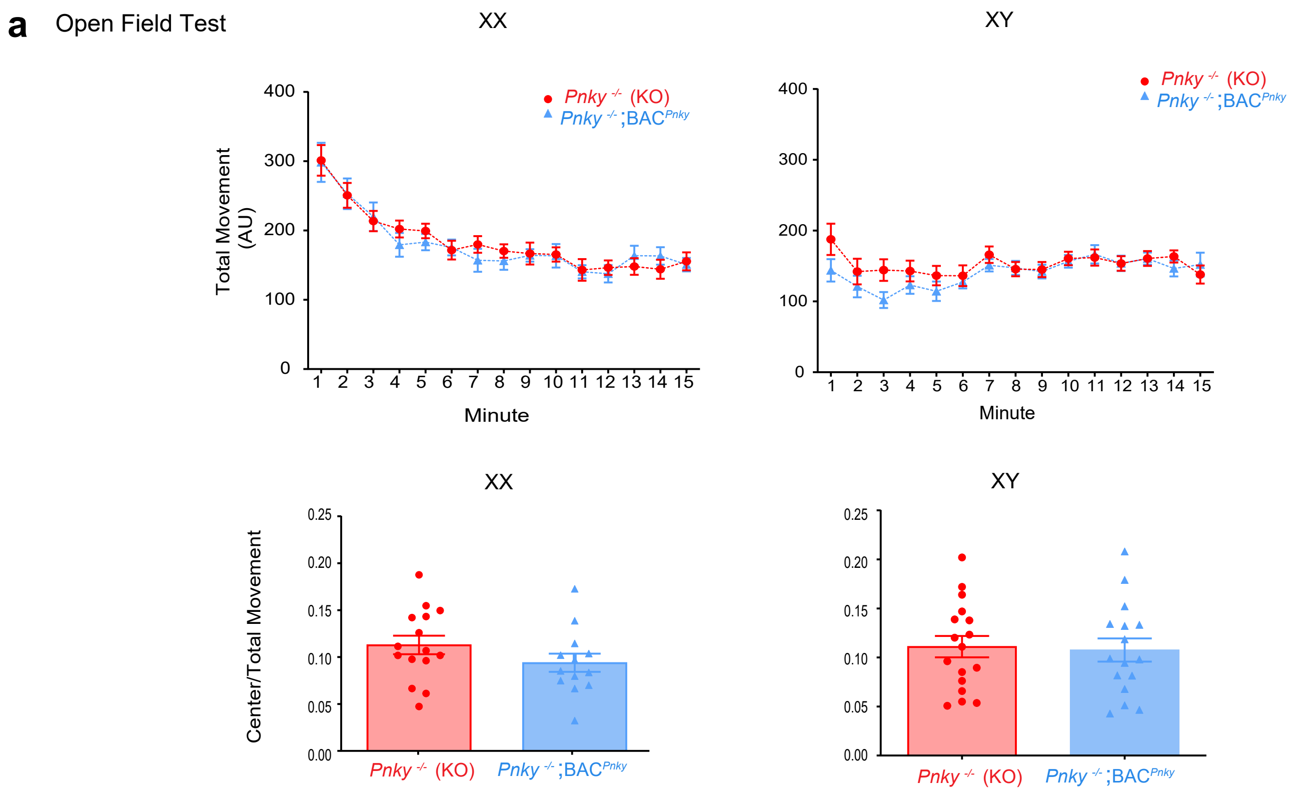

Supplementary Fig. 7: Open field test *Pnky*-KO vs *Pnky*-KO;BAC-*Pnky*. a) In the OFT, no significant differences between XX and XY mice of *Pnky*-KO and *Pnky*-KO; BAC-*Pnky* groups in total movement (repeated measures two-way ANOVA, p = ns ; XX: p = 0.8135 and XY: p = 0.3404) and ratio of center to total movement; two-tailed unpaired Welch's t tests, p = ns (XX: p = 0.1819 , XY: p = 0.8986). n = 15 XX and 18 XY *Pnky*-KO mice and n = 13 XX and 16 XY *Pnky*-KO;BAC-*Pnky* mice. Data is represented as mean ± SEM. Source data are provided as a Source Data file.

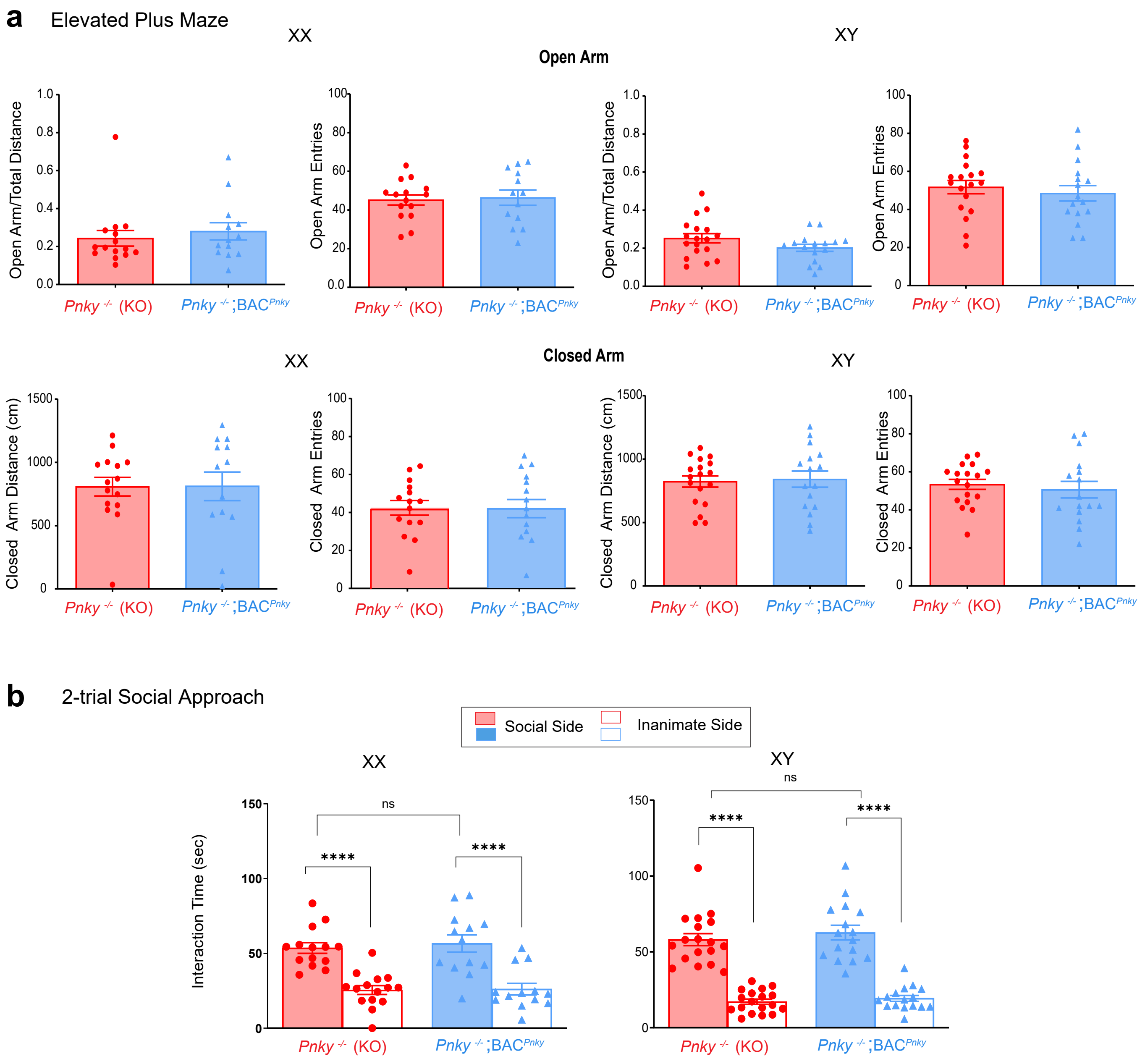

Supplementary Fig. 8: Elevated Plus Maze and Social approach test *Pnky*-KO vs *Pnky*-KO;BAC-*Pnky*. a) In the EPM, the ratio of open arm distance to total distance (XX, Mann-Whitney, *p* = ns (0.3874); XY, Welch's *t* test, *p* = ns (0.1110)), number of open arm entries (Welch's *t*-tests, *p* = ns (XX, p = 0.8052; XY, p = 0.5467)), number of closed arm entries (Welch's *t* tests, *p* = ns (XX, p = 0.9512; XY, p = 0.5923)) and closed arm distance (Welch's *t* tests, *p* = ns (XX, p = 0.9863; XY, p = 0.8119) are comparable between the *Pnky*-KO and *Pnky*-KO; BAC-*Pnky* groups.
b) XX and XY mice of both genotypes show increased preference (interaction time in seconds) for the social side in the 2-trial social approach test whereas the difference in interaction time across genotypes is non-significant. Paired *t* test ****p= 0.0001 for social vs inanimate side for both sexes for both *Pnky*-KO and *Pnky*-KO; BAC-*Pnky* groups. Welch's *t* test *p* = ns (XX, *p* = 0.6633; XY, *p* = 0.4633 for *Pnky*-KO vs *Pnky*-KO; BAC-*Pnky* social interaction time. ****p <0.0001, ns = non-significant. n = 15 XX and 18 XY *Pnky*-KO mice and n = 13 XX and 16 XY *Pnky*-KO;BAC-*Pnky* mice. All Mann-Whitney, Welch's *t* tests and paired *t* tests are two tailed. Data is represented as mean ± SEM. Source data are provided as a Source Data file.

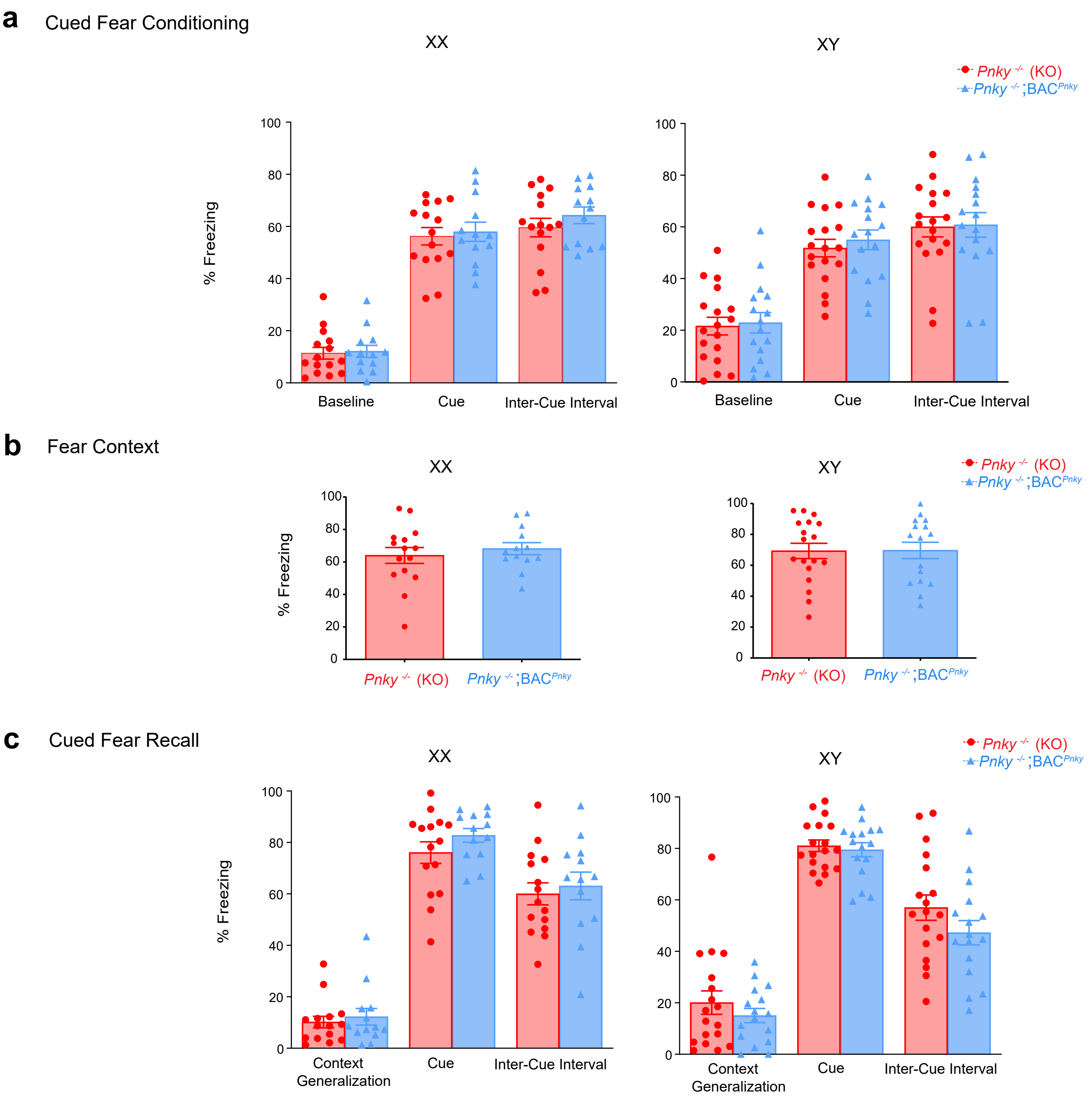

Supplementary Fig. 9: Cued fear conditioning and recall test *Pnky*-KO vs *Pnky*-KO;BAC-*Pnky*. a) XX and XY mice of *Pnky*-KO and *Pnky*-KO; BAC-*Pnky* groups show no significant difference in cued Fear conditioning (multiple t tests (Holm-Šídák method) for the baseline, cue presentations and ICI for XX and XY mice, p = ns (Baseline- XX: $p = 0.8258$, XY: $p = 0.8100$; Cued XX: $p = 0.7341$, XY: $p = 0.5380$; ICI- XX: $p = 0.3364$, XY: $p = 0.9001$). **b)** fear context recall (two-tailed, Welch's t test, p = ns; XX: $p = 0.5064$ and XY: $p = 0.9624$), and **c)** cued fear recall tests multiple t tests (Holm-Šídák method) for the context generalization, cue presentations and ICI for XX and XY mice, p = ns (Generalization- XX: $p = 0.5926$, XY: $p = 0.3647$; Cued- XX: $p = 0.2043$, XY: $p = 0.6598$; ICI- XX: $p = 0.6573$, XY: $p = 0.1648$). n = 15 XX and 18 XY *Pnky*-KO mice and n = 13 XX and 16 XY *Pnky*-KO;BAC-*Pnky* mice. Data is represented as mean ± SEM. Source data are provided as a Source Data file.

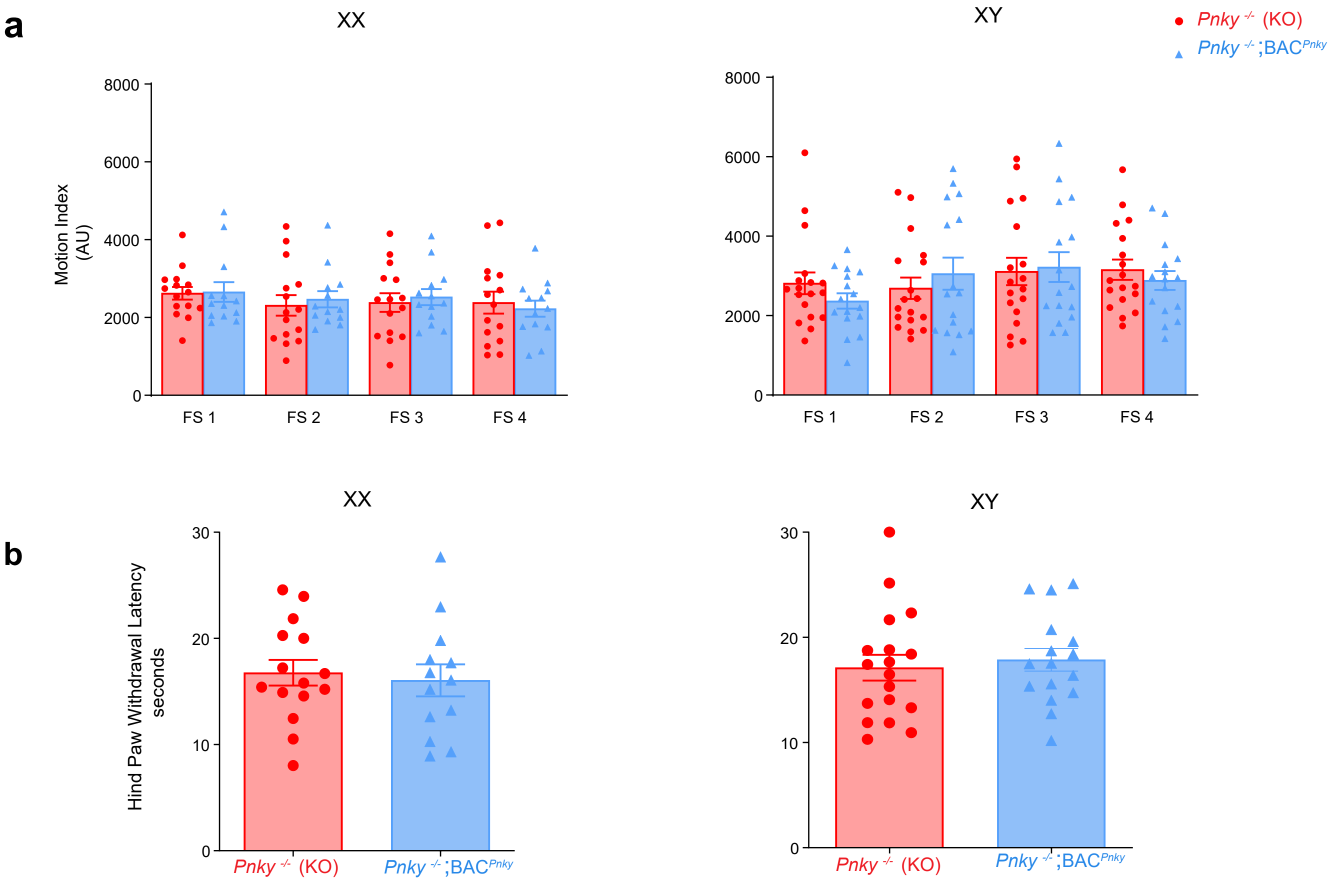

Supplementary Fig. 10: Motion Index and Hind paw withdrawal latency test *Pnky*-KO vs *Pnky*-KO ;BAC-*Pnky*. a) In the Motion index test of the Cued Fear conditioning, no significant differences between *Pnky*-KO vs *Pnky*-KO; BAC-*Pnky* XX and XY mice; Multiple *t* tests, p = ns (XX: Shock 1 $p = 0.4243$, Shock 2 $p = 0.7305$, Shock 3 $p = 0.0711$, Shock 4 $p = 0.7466$ and XY: Shock 1 $p = 0.8439$, Shock 2 $p = 0.6604$, Shock 3 $p = 0.9966$, Shock 4 $p = 0.0509$). **b)** *Pnky*-KO vs *Pnky*-KO; BAC-*Pnky* mice showed no significant difference in hind paw withdrawal latency during the hot-plate test. (For XX genotype two-tailed Welch's *t* test $p = 0.7144$; for XY genotype two-tailed Mann-Whitney test, $p = 0.5738$). n = 15 XX and 18 XY *Pnky*-KO mice and n = 13 XX and 16 XY *Pnky*-KO;BAC-*Pnky* mice. Data is represented as mean ± SEM. Source data are provided as a Source Data file.

a Pre-pulse Inhibition

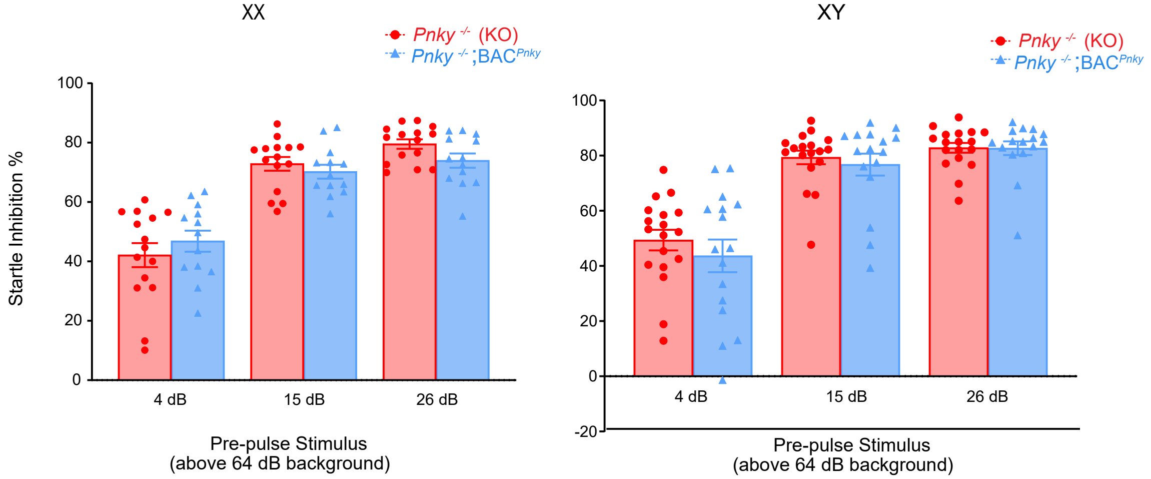

Supplementary Fig. 11: Pre-pulse inhibition *Pnky*-KO vs *Pnky*-KO ;BAC-*Pnky*. a) *Pnky*-KO and *Pnky*-KO; BAC-*Pnky* animals exhibit no significant changes in the startle inhibition at prepulse intensities; multiple *t* tests (Holm-Šídák method) for each sex, *p* = ns (4dB (XX: *p* = 0.3953 XY *p*= 0.4104), 15dB (XX: *p* = 0.4262 XY *p* = 0.5772) and 26dB (XX: *p* = 0.0584 XY: *p* = 0.9582)) above the background. n = 15 XX and 18 XY *Pnky*-KO mice and n = 13 XX and 16 XY *Pnky*-KO;BAC-*Pnky* mice. Data is represented as mean ± SEM. Source data are provided as a Source Data file.

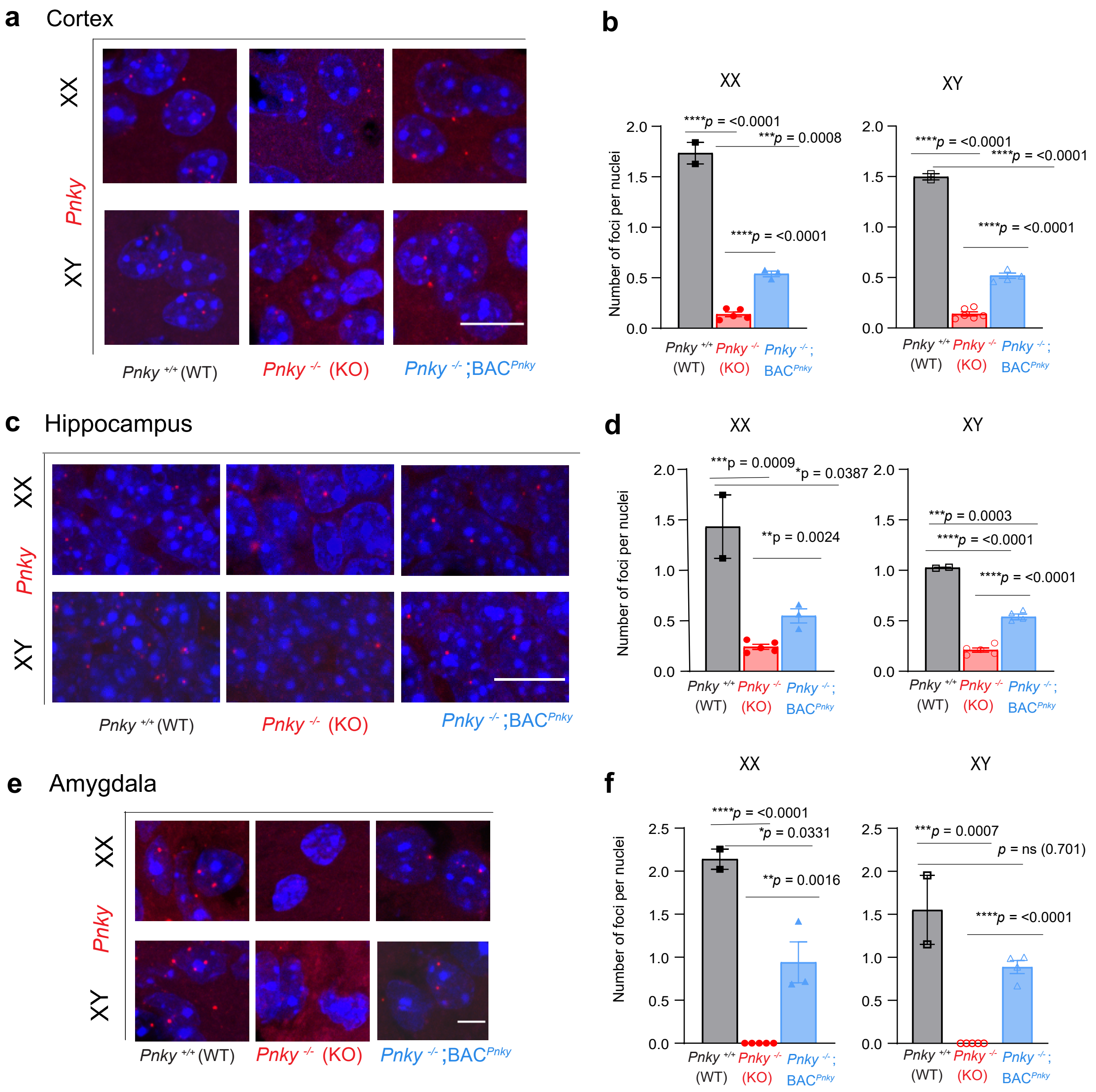

Supplementary Fig. 12: RNA fluorescent in-situ hybridization on fixed-frozen brain sections to assess *Pnky* RNA expression in **a)** cortex, **c)** hippocampus (Scale bar = 10 µm) and **e)** amygdala (Scale bar = 5 µm). DAPI was used to label nuclei. Quantification of RNAScope *Pnky* signal (red puncta) presented as Number of foci per nuclei in **b)** cortex (100-120 nuclei) **d)** hippocampus (100-150 nuclei) and **f)** amygdala (20-40 nuclei) were counted across 3-4 non-overlapping ROIs of fixed area per animal (n= 2-6 animals per sex and genotype). unpaired *t* test (two-tailed) was used and data is represented as mean ± SEM. ns = not significant. *p <0.05, **p < 0.01, ***p < 0.001, ****p < 0.0001. Source data are provided as a Source Data file.
