# Supplementary material for: Sex-specific role for the long noncoding RNA Pnky in mouse behavior
Source: Nat Commun. 2024 Aug 12;15:6901. doi: 10.1038/s41467-024-50851-7 (PMC11319455; doi:10.1038/s41467-024-50851-7)
Supplement: Supplementary file 3 — Description of additional supplementary files [file 41467_2024_50851_MOESM3_ESM.pdf]

<u>Description of Additional Supplementary Files</u>

File name: Supplementary Data 1

Description: Behavioral tests data generated in this study. Supplementary Data 1 contains the data from all the behavioral tests in this study for two cohorts (*Pnky*-WT vs *Pnky*-KO and *Pnky*-KO vs *Pnky*-KO;BAC-*Pnky*) of animals.
