# Supplementary material for: Sex-specific role for the long noncoding RNA Pnky in mouse behavior
Source: Nat Commun. 2024 Aug 12;15:6901. doi: 10.1038/s41467-024-50851-7 (PMC11319455; doi:10.1038/s41467-024-50851-7)
Supplement: Supplementary file 4 — Supplementary Data 1 [file 41467_2024_50851_MOESM4_ESM.zip › UnblindedGenotype.docx]

Blinded genotype

Cohort 1 : *Pnky*-WT and *Pnky*-KO

A – *Pnky*-WT female , B – *Pnky*-KO female, C – *Pnky-*WT male and D- *Pnky-*KO male.

Cohort 2 : *Pnky*-KO and *Pnky*-KO; BAC-*Pnky*

A – *Pnky*-KO female , B - *Pnky*-KO; BAC-*Pnky* female, C - *Pnky*-KO male and D- *Pnky*-KO; BAC-*Pnky* male.
